# Correcting Inconsistencies and Errors in Bacterial Genome Metadata Using an Automated Curation Tool in Excel (AutoCurE)

**DOI:** 10.3389/fbioe.2015.00138

**Published:** 2015-09-11

**Authors:** Sarah E. Schmedes, Jonathan L. King, Bruce Budowle

**Affiliations:** ^1^Institute of Applied Genetics, Department of Molecular and Medical Genetics, University of North Texas Health Science Center, Fort Worth, TX, USA; ^2^Center of Excellence in Genomic Medicine Research, King Abdulaziz University, Jeddah, Saudi Arabia

**Keywords:** bacteria, genomes, metadata, database, curation, automation, AutoCurE

## Abstract

Whole-genome data are invaluable for large-scale comparative genomic studies. Current sequencing technologies have made it feasible to sequence entire bacterial genomes with relative ease and time with a substantially reduced cost per nucleotide, hence cost per genome. More than 3,000 bacterial genomes have been sequenced and are available at the finished status. Publically available genomes can be readily downloaded; however, there are challenges to verify the specific supporting data contained within the download and to identify errors and inconsistencies that may be present within the organizational data content and metadata. AutoCurE, an automated tool for bacterial genome database curation in Excel, was developed to facilitate local database curation of supporting data that accompany downloaded genomes from the National Center for Biotechnology Information. AutoCurE provides an automated approach to curate local genomic databases by flagging inconsistencies or errors by comparing the downloaded supporting data to the genome reports to verify genome name, RefSeq accession numbers, the presence of archaea, BioProject/UIDs, and sequence file descriptions. Flags are generated for nine metadata fields if there are inconsistencies between the downloaded genomes and genomes reports and if erroneous or missing data are evident. AutoCurE is an easy-to-use tool for local database curation for large-scale genome data prior to downstream analyses.

## Introduction

Advancements in sequencing technologies in the past several years have resulted in a substantial increase in the number of bacterial genomes that have been and continue to be sequenced. The first complete bacterial genome was sequenced in 1995 (Fleischmann et al., 1995) and 24 microbial organisms were completely sequenced within the next 5 years (Nierman et al., 2000). Ten years later, in 2005, there were almost 300 prokaryote genomes sequenced (Fraser-Liggett, 2005) and as of May 2015 there were 34,066 bacterial genomes available at the complete (3,725), chromosome (773), scaffold (11,028), and contig (18,540) status as listed by the National Center for Biotechnology Information (NCBI)^1^. Integrated Microbial Genomes (IMG)^2^ (Markowitz et al., 2012) reported the number of bacterial genomes at 26,033 at the finished (3,378), draft (1,683), and permanent draft (20,972) status, and there is a total of 39,969 bacterial genome sequencing projects listed in the Genomes OnLine Database (GOLD)^3^ (Reddy et al., 2015), an increase from only 1,986 in 2007. As a result of advancements in sequencing technologies, with increased output and decreased costs, the number of completed genomes will continue to rise resulting in substantial amounts of data.

These whole bacterial genome sequence data are housed in publically available databases such as NCBI^4^ (Benson et al., 2015), European Molecular Biology Laboratory–European Bioinformatics Institute (EMBL–EBI)^5^ (Amid et al., 2012), and DNA Data Bank of Japan (DDBJ)^6^ (Kodama et al., 2012), which make up the International Nucleotide Sequence Database Collaboration (INSDC) (Nakamura et al., 2013). Additional databases with more specific microbial applications and bioinformatics programs include IMG (Markowitz et al., 2012) and PATRIC (Pathosystems Resource Integration Center) (Wattam et al., 2014). Data can be readily downloaded from these databases through ftp sites or facilitated through download links. The NCBI ftp site^7^ provides links to download all bacterial genomes in a number of file types. However, since these downloads include thousands of complete bacterial genomes, there is a challenge to easily identify which genomes were included in the download, to determine if all files and metadata associated with particular genomes were included and whether supporting data were correct. Quality control of supporting data within public databases is crucial to ensure accurate and the most up-to-date metadata; however, quality control practices and methods are not readily known or clearly stated. Inaccurate identifying information can confound downstream analyses and may cause misinterpretations and therefore curation of metadata is necessary. High-quality databases are essential for research areas, such as comparative genomics, phylogenetics, and metagenomics, especially as they apply to diagnostics, public health, biosafety and biosecurity, and microbial forensics.

In this study, a local database was created that contained all publically available complete bacterial genomes from the NCBI ftp site. Metadata inconsistencies were observed between the downloaded genomes and those listed as complete genomes on the genome reports from NCBI Genome. To use these data for downstream studies, a manual curation was performed to identify and correct inconsistencies and to delete erroneous files. Manual curation was performed to compare the supporting data associated with each sequence file, including genome name, UID (unique identifier) number, RefSeq accession numbers, and file descriptions found within each file to the metadata included in the complete genome reports. The process was performed using a “one-by-one” approach which was time consuming and not routinely practical for future efforts, especially as the number of genome entries continues to increase. Therefore, an automated tool for bacterial database curation in Excel (AutoCurE)^8^ was developed, decreasing the curation time from months with manual curation to minutes with automated curation. AutoCurE facilitates checks between the downloaded genome folders, files and the genome reports to flag if any inconsistencies exist in the metadata, including genome names, BioProject/UID, RefSeq accession numbers, and sequence file descriptions, and to identify and flag archaea genomes.

## Materials and Methods

### Genomes

All complete bacterial genomes were downloaded on March 5, 2014 from the Bacteria folder on the NCBI ftp site^7^ using the all.fna.tar.gz link to retrieve all fna files (DNA genome sequence in FASTA format). Genome reports of all complete bacteria and archaea genomes were downloaded from NCBI Genome^9^ on March 6, 2014. No modification dates were listed on the genome reports for March 5, 2014 to March 6, 2014 (to rule out discrepancies between the genome file and genome report download dates).

### Manual curation

Manual curation of the local bacterial genomes database was performed in three rounds. In Round 1, downloaded genome folder names were compared with the complete bacterial and archaeal genome reports to identify archaea genomes and bacterial genomes found in the genome report by name. In Round 2, genomes not found on either report were searched by RefSeq accession numbers from the files to identify genomes on reports that had been renamed. Any remaining genomes still not found on the genome reports were searched on NCBI to determine if the file had been discontinued or to verify the identity of the genome. In Round 3, “one-by-one” manual curation was performed to check genome names and files against the genome reports at the time of download in addition to current information on NCBI for genome name, BioProject/UID, file description, and RefSeq accession numbers.

### AutoCurE development and features

AutoCurE was developed to provide an automated approach for bacterial database curation of downloaded supporting data from fna file types from the NCBI ftp site. AutoCurE is composed of two customized Excel workbooks, the AutoCurE Genome Filename Tool and the AutoCurE Genome Report Tool, with custom scripts and macros to: facilitate creating a print directory and file path of all downloaded genomes; rename all file names to the first line of text (to make the files more recognizable as opposed to just providing the accession number); parse out metadata fields to facilitate searches; and create flags to mark inconsistencies or errors between the downloaded genome files and the current bacteria and archaea genome reports. Flags are generated for nine different metadata categories to identify inconsistencies or errors pertaining to the following: (1 and 2) genome name for genus and species (strain was not included due to the wide variation of naming inconsistency of strain names); (3) to identify archaea; (4) to verify consistency between the original filename accession number and the accession number found within each sequence file; (5) to identify inconsistencies between the UID number from the genome folder name and the BioProject ID within the genome report; (6) identify if the RefSeq accession number from each sequence file is found within the genome report; (7) identify accession numbers other than RefSeq reference assembly accessions (i.e., other than NC_XXXXXX); (8) identify genome folders missing whole genome or chromosome files (i.e., only contains plasmid files); and (9) identify sequence files which may be a draft sequence. Report statements are generated for each flag to notify the user of potential changes or corrections that need to be made. AutoCurE also facilitates file manipulation by allowing the user to select sets of specific genomes and copies of the files are moved to a new directory to maintain an unaltered master copy of the database. This feature eliminates having to manually search and retrieve files for downstream use. All processing times reported were using a computer with i7-2600 CPU @ 3.4 GHz, 3.23 GB of RAM.

## Results

### Manual curation

All complete genomes in the Bacteria folder on the NCBI ftp site (*N* = 2,769; downloaded March 5, 2014) were downloaded to create a local bacterial genome database. Genome names from each of the genome folders were compared with the genome reports to separate bacteria genomes from archaea genomes (Table 1). Archaea genomes (*N* = 164) were found within the Bacteria folder and were removed. In addition, 157 genomes were not found on either report by genome name. In order to verify the identity of these genomes, RefSeq accession numbers (as listed as the sequence file names) were searched against each genome report. Of these genomes searched, 87 bacterial genomes were found on the genome report associated with a different genome name; 59 bacterial genomes and 5 archaeal genomes were not found on the genome report but were found in the NCBI Nucleotide database; and 6 bacterial genomes had been removed by NCBI, and the accession numbers had been discontinued.

**Table 1:** **Inconsistencies between genome downloads and genome reports**.

		Bacteria	Archaea	Total
Round 1 (genome name search)	Downloaded genomes from ftp site Complete genomes listed in genome report Downloaded genome names found within report Not found in genome report by name	2,605 2,734 2,453	164 168 159^a^	2,769 2,902 2,612 157
Round 2 (genome accession number search)	Accession number found in genome report, genome name change (includes strain name) Accession numbers discontinued Accession number not found in genome report but found on NCBI Nucleotide	87 6 59	5	64 2,599
Round 3 (“one-by-one” manual curation)	Starting number of genomes No inconsistencies or errors observed Not found on genome report Found on genome report but no accession numbers listed Genus and/or species name inconsistent Potential draft sequence Chromosome/genome data missing (only plasmid files present) Changed from complete status Genome folder contained erroneous files Genomes deleted for not containing complete reference assemblies Final number of bacterial genomes in local database	2,402 57 18 68 56 5 131 9 19		2,580

*^a^Kra 1 was found in both the bacteria and archaea genome reports*.

Although the majority of the genome folders (*N* = 2,402) were named correctly and contained the correct files, other types of errors and inconsistencies were observed. These problematic data included duplicate genome names, non-reference assembly file types (i.e., contig or scaffold files, environmental sequence files, and genome folders only containing plasmids), naming inconsistencies, and files misplaced in genome folders. In order to verify all files and associated metadata, a “one-by-one” manual curation was performed on 2,599 bacterial genomes in the database after all archaea and discontinued genomes had been removed. Downloaded genome folder names, BioProject/UID numbers, and sequence file descriptions and accession numbers (first line of text within fna files) were compared with the metadata included in the genome reports to identify inconsistencies. The most common discrepancies were inconsistent genome name nomenclature between the genome folder name, genome report, and within the fna file (*N* = 68; genus and species names), indicating inconsistent updates when genome names are changed. For example, Candidatus *Endolissoclinum faulkneri* L2, BioProject PRJNA182483, had a genome folder named *Thalassobaculum* L2 containing an fna file with the genome name Candidatus *Endolissoclinum patella* L2; thus, illustrating the discrepancies that can occur between the genome report, genome folder, and fna file. Inconsistent genome names also included likely major spelling errors. In addition, there were examples of genome folders having the same name, including strain (*N* = 83), with the only differences in the accession numbers and BioProject IDs. The bacteria genome report contained 126 duplicate genome names, 7 of which had duplicate BioProject IDs, and 64 of the duplicate genome names were associated with the 83 duplicate genome folder names. While duplicate genome names were not considered errors, as these are the correct names, it does point out the need for better naming requirements (such as substrain or isolate ID) to differentiate another genome from another in addition to solely the BioProject ID. In addition, 57 genomes were not found on the genome report but were found on NCBI Nucleotide, and 18 genomes were found on the report but had no accession numbers associated with the genome. Additionally, 19 genome folders were removed due to RefSeq accession numbers for associated fna files being discontinued and removed from NCBI, RefSeq accession numbers not listed on genome reports or genome page, genome status changed to scaffold-level, genome folder only contained plasmid files, and not all chromosome files were included in genome folder, resulting in 2,580 genomes in the local database. Round 3 manual curation includes the results found within Round 2, and the results are more inclusive.

Erroneous files were also found within the downloaded data and associated with incorrect genomes. The downloaded data were retrieved from the complete bacterial genomes folder on the NCBI ftp site; however, 56 genomes were found as potential draft genomes (i.e., text within fna files listed these sequences as “draft,” “partial sequence,” “provisional sequence,” “nearly complete genome,” “sequencing in progress,” and “non-contiguous finished genome”), 5 genome folders only contained plasmid files, and in the course of manual curation, 3 genomes were changed to scaffold status and 128 genomes changed from “complete” to “chromosome” or “chromosome with gaps” status. In addition, nine genome folders contained erroneous files which either did not belong to that particular genome or were not RefSeq reference assembly files. For example, the genome folder for *Vibrio parahaemolyticus* O1 K33 CDC K4557 contained the two correct chromosome files for this genome; however, an additional 17 files were found within the genome folder belonging to a different strain of *Vibrio parahaemolyticus*, 9 different strains of *Listeria monocytogenes*, and 1 strain of *Campylobacter jejuni*. Additionally, at the time of download, there were six different substrains of *Synechocystis* sp. PCC 6803, of which three substrains, including GT-I, PCC-N, and PCC-P, had incorrectly associated substrain names, BioProject/UIDs, and fna files.

### AutoCurE

AutoCurE was developed to automate curation of supporting data of local bacterial genome databases from data downloaded from the NCBI ftp site. AutoCurE is composed of two Excel workbooks, the AutoCurE Genome Filename Tool and the AutoCurE Genome Report Tool, with customized scripts to automatically generate flags for nine different metadata categories to identify inconsistencies and errors of the types found during manual curation, which are listed in Table 2. After whole-genome data and genome reports are downloaded, AutoCurE generates print lists of the genomes downloaded and compares this list to the bacteria and archaea genome reports to identify archaea and compare the genome name, BioProject/UIDs, RefSeq accession numbers, and fna sequence file descriptions to flag inconsistencies between the downloaded data and genome reports. Report statements are generated for each flag, notifying the user of corrections that may need to be made to the local database.

**Table 2:** **AutoCurE Genome Filename and Report Tools**.

**Features**
Prints list directory of downloaded genomes and file paths
Pulls out first line of text from files to provide RefSeq accession number and sequence file description
Parses metadata from genome reports and data downloads into lists to compare BioProject/UID, RefSeq accession number, genome folder name, file name, and file description
File manipulation to eliminate manual searching within directories. Allows the user to check desired genomes in the Excel workbook and a copy of the genome files is made to another directory for downstream use, thus keeping an unaltered master copy of the database
**Flags**
*Accession number in genome report*: indicates if the accession number within the sequence file is found in the genome report
*BioProject/UID match*: compares the UID from downloaded genome folder names to BioProject ID in the genome report
*Original accession consistency*: indicates if the original accession number file name matches the accession number found within the sequence file
*Genus match*: compares genus name from genome report to genome folder to sequence file
*Species match*: compares species name from genome report to genome folder to sequence file
*Archaea*: identifies archaea genomes based on accession numbers found in the archaea genome report
*RefSeq reference genome accession*: identifies any files with an accession number other than a RefSeq reference assembly number
*Chromosome/genome data present*: indicates if only plasmid sequence files are present (i.e., chromosome or whole-genome data are absent)
*Draft or partial sequence*: identifies any potential draft genome or partial sequences based on sequence file description

**Figure 1:**
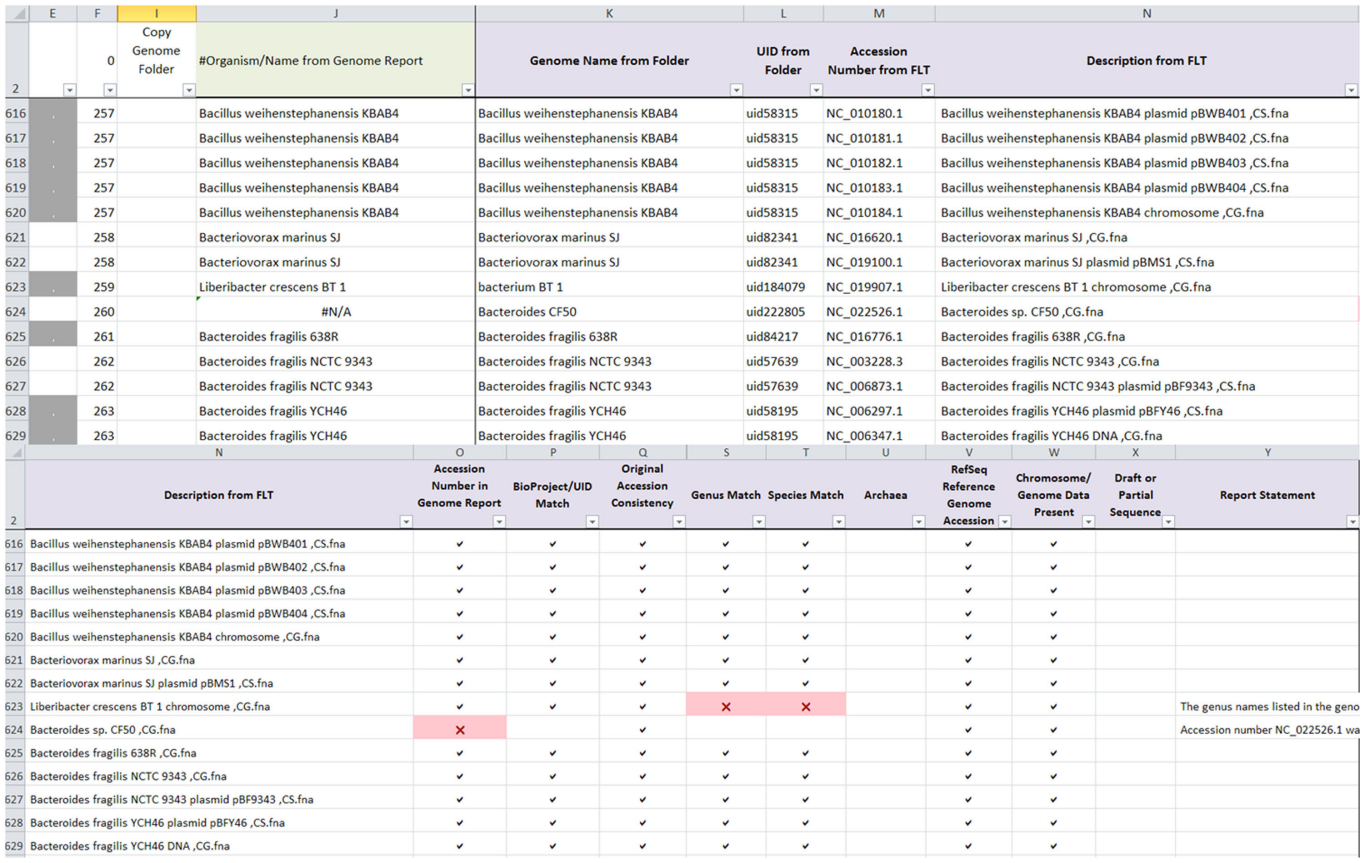
**AutoCurE Genome Report Tool**. AutoCurE compared content from the genome report, genome folder name, and fna file description to flaginconsistencies for nine metadata categories. Flags, shown as red Xs, were generated, indicating that a RefSeq accession number was not found in the genome report and inconsistencies in genus and species name. Additional columns in the Genome Report Tool, not shown, include a Comments section and metadata taken from the NCBI genome reports associated with each downloaded file. Columns E and F group the files associated with a particular genome by color (Column E) and by number (Column F). (FLT, First Line of Text within the fna file).

The same genome dataset from Round 2 manual curation was used to validate the ability of AutoCurE to compare the automated results with the manual curation results. In addition, 10 archaea genomes were included to validate the archaea flag, since all known archaea found on the archaea genome report had been removed prior to the Round 2 genomes dataset. AutoCurE processed 2,621 genomes (4,956 files) in less than 30 min. In comparison, manual curation took several months (with a 2–3 days per week effort). By default, AutoCurE can process up to 10,000 files; however, more genomes/files can be easily accommodated with minor formula modifications. Flags were successfully generated for each of the nine categories. Figure 1 shows an example of the AutoCurE Genome Report Tool flagging multiple fna files in the accession number and genus and species name categories. Report statements were generated for each flag, indicating potential changes which need to be made to the database files or metadata. Each flag was manually checked to ensure that flags were appropriately generated.

The total number of flags produced for each category by AutoCurE was consistent with values from manual curation with a few discrepancies. Discrepancies between the manually curated dataset and the AutoCurE dataset include genome name inconsistencies in the species name category when a species name is not listed or when inconsistent punctuation may be present. A number of genus and species inconsistencies were also observed due to minor spelling errors (one letter difference). Since flags are generated based on customized formulas, anything outside the search parameter may be missed. For example, *Fibrella aestuarina* BUZ 2 genome was not flagged as a potential draft sequence due to a spelling error in the sequence file description, “drat genome.” In addition, since *Thermoproteus tenax* Kra 1 was listed on both the bacteria and archaea genome reports but only had an accession number listed in the bacteria genome report, AutoCurE did not mark this genome as archaea, due to this error. Any errors within the genome report will not be flagged; only inconsistencies between the downloaded data and genome reports will be identified. Additionally, the File Management Center within the Genome Report Tool, which incorporates the file manipulation feature, was validated. More than 4,000 files were copied and moved to an output directory in less than 15 min. Smaller file batches (*N* = 50) can be moved in about 1 sec.

## Discussion

Whole-genome data are available at a number of public repositories. Some of these data are not necessarily curated, constantly being updated, and in flux. Therefore, it is expected that errors and inconsistencies will arise, such as in genome names, since taxonomic name changes occur frequently. One should be aware of the types of inconsistencies and errors that are and may be present in order to correct them before using the data for research and development in diagnostics, public health, biosafety and biosecurity, and microbial forensics. In this study, inconsistencies and errors were observed while creating a local bacterial genome database using whole-genome data available from the NCBI ftp site. The main issues observed included: archaea genomes were colocalized in the same folder as the bacteria genomes; genome naming inconsistencies were observed between the genome folders, genome reports, and fna files; not all data downloaded were included in the genome reports and not all genomes found on the genome reports were available for download on the ftp site; discontinued files had not been removed from the ftp site; some genome folders contained draft genome or only plasmid files; and files were associated with incorrect genomes. In addition, during the course of manual curation, more than 130 genomes had been changed from “complete” to “chromosome,” “chromosome with gaps,” or “scaffold” status, indicating fluidity in genome status as genomes are updated; because of this lack of consistency, official genome status should be checked in GOLD (Reddy et al., 2015). Due to discrepancies in downloaded data from genome databases, proper curation is necessary prior to downstream use to reduce misinterpretations that may affect subsequent analyses.

As the number of available genomes continues to increase, it will not be practical to manually curate data. To reduce errors that may impact subsequent analyses, it is imperative to curate the downloaded data contained within local databases to remove redundancies, erroneous files, and correct for naming inconsistencies. An automated tool was needed to authenticate supporting data associated with downloaded publically available bacterial genomes. AutoCurE was developed to facilitate curation of local bacterial databases by reducing curation time from months to minutes while automatically flagging errors and inconsistencies. Other tools have been developed for local database storage and manipulation, such as MicrobeDB (Langille et al., 2012). MicrobeDB is a Linux-based database tool facilitating genome downloads from the NCBI ftp site, archiving files and updating the database, and database manipulation (Langille et al., 2012). While a useful tool for bacterial database manipulation, MicrobeDB requires the user to be familiar with Perl programming or with MySQL (Langille et al., 2012). In contrast, AutoCurE is Excel-based to provide ease-of-use in a Windows-based platform for metadata curation and database manipulation, which may be better suited for users not adept at programming.

There is a need for better quality checks as databases are maintained and updated to check for naming inconsistencies/changes, updating sequence files, removing discontinued files, and checking for correct file placement and UID associations. Recommendations and changes have been made by the INSDC to replace genome identifiers from strain taxids with alternative, more unique metadata, such as BioSample, BioProject, or assembly ID (Federhen et al., 2014). Moving toward a metadata system of more unique identifiers helps reduce ambiguities when genomes are named with the same strain name. However, improved quality control of database management needs to be implemented to maintain the most up-to-date and accurate files and metadata on public repository sites. AutoCurE provides a solution for automated curation of these supporting data to provide a quality check prior to using the downloaded files, thus eliminating the need for manual curation or downloading each genome one at a time. Improved upfront quality control of data directly by public database managers would reduce the need for downstream resources and provide a seamless flow of higher quality data and metadata directly to the end user. As genome data continue to grow, quality control practices and additional tools, such as AutoCurE, are exceedingly important for data storage, curation, and manipulation.

## Author Contributions

SS and BB conceived the project. SS and JK designed and developed the analytical tools. SS analyzed the data. SS, JK, and BB wrote the article.

## Conflict of Interest Statement

The authors declare that the research was conducted in the absence of any commercial or financial relationships that could be construed as a potential conflict of interest.

## Funding

This project was supported by internal funds from the Institute of Applied Genetics, University of North Texas Health Science Center.

## Footnotes

^1^http://www.ncbi.nlm.nih.gov/genome/browse/, accessed May 28, 2015

^2^https://img.jgi.doe.gov/cgi-bin/m/main.cgi?section=ImgStatsOverview, accessed May 28, 2015

^3^https://gold.jgi-psf.org/statistics

^4^http://www.ncbi.nlm.nih.gov/genbank/

^5^http://www.ebi.ac.uk/ena

^6^http://www.ddbj.nig.ac.jp/

^7^ftp://ftp.ncbi.nlm.nih.gov/genomes/Bacteria/

^8^AutoCurE is available and maintained by the Institute of Applied Genetics at https://www.unthsc.edu/graduate-school-of-biomedical-sciences/molecular-and-medical-genetics/laboratory-faculty-and-staff/AutoCurE/. AutoCurE will be updated based on user feedback and any changes made to genome report formats and/or structure by NCBI.

^9^http://www.ncbi.nlm.nih.gov/genome/
